# Comparative evaluation of therapeutic interventions during hemorrhagic shock

**DOI:** 10.1186/cc9510

**Published:** 2011-03-11

**Authors:** D Fantoni, DA Otsuki, AR Martins, JA Filho, E Andrades, E Chaib, FA Voorwald

**Affiliations:** 1 USP, São Paulo, Brazil; 2 FCAV/UNESP, Jaboticabal, Brazil

## Introduction

Resuscitation of patients with hemorrhagic shock (HS) represents a challenge in emergency medicine. The uncontrollable bleeding and subsequent cardiovascular collapse are responsible for 40% of the early mortality rate in trauma.

## Methods

Twelve Large White pigs at 5 months of age, weighing 25 kg, were submitted to a surgical procedure for liver resection or autologous liver transplantation. Ketamine S+ (5 mg/kg, i.m.) and midazolam (0.5 mg/kg, i.m.) were used as a premedicant. Anesthesia was induced with propofol (3 mg/kg, i.v.) and maintained with 1.5% isoflurano end-tidal concentration and volume-controlled ventilation (8 ml/kg) on 40% inspired oxygen fraction. Analgesia and neuromuscular blockade were accomplishments with continuous infusion of fentanyl (0.4 mg/kg/minute) and pancuronium (0.3 mg/kg/hour). The shock was diagnosed when blood loss exceeds 40% of the total blood volume. The HS results in mean arterial pressure reduce (MAP ≤50 mmHg), 50% cardiac output reduction (CO) and central venous saturation (SvO_2_) decreased to 70 mmHg. The animals underwent hemodynamic, arterial blood gases and venous monitoring, at baseline (t0), impact moment (t1), after treatment (t2), intervals of 15 minutes after shock treatment (t3, t4, t5, t6), and 120 minutes after treatment (t7). Subsequent to shock diagnosis, the animals were randomly divided into GI treated with vasopressin (0.01 IU/kg/minute), norepinephrine (0.3 mg/kg/minute) and Ringer's lactate solution (aliquots of 20 ml/kg/20 minutes until MAP >60 mmHg). GII was equal to GI but ringer lactate administration was replaced during 20 minutes of whole blood stored during 10 days at half blood loss volume.

## Results

See Table 1. Both groups showed a significant parameter decrease during hemorrhagic shock (t1) compared with t0. After treatment GI showed improvements in all parameters, GII showed improvement until t3. During t4 the animals presented a significant increase in K levels, lactate and decreased SvO_2_, CO, MAP followed by an increase in SvO_2 _(89%). The differences between the two groups and moments were statistically significant (*P *> 0.01). GII had a 50% of mortality rate between t4 and t5 related with potassium increase. Subsequent to animal blood treatment, the patients showed an increase in T wave, ventricular fibrillation and death.

**Table 1:** 

	CO	MAP	**SvO**_**2**_	PAP	K	Lactate
	(l/min)	(mmHg)	(%)	(mmHg)	(mmol/l)	(mg/dl)
t0 GI	3.6 ± 0.4	86 ± 10	75 ± 3	18 ± 2	3.5 ± 0.4	19 ± 8
t1 GI	1.3 ± 0.3	48 ± 10	58 ± 5	8 ± 3	4 ± 0.3	47 ± 8
t0 GII	4 ± 0.4	84 ± 8	76 ± 3	20 ± 3	4 ± 0.2	20 ± 10
t2 GII	1.5 ± 0.5	44 ± 5	57 ± 3	10 ± 2	4.3 ± 0.3	50 ± 108

## Conclusions

It is possible to conclude that whole blood replacement in animals with HS should be slow and steady to avoid the effects of high K administration during a short period. Those therapeutic interventions are indicated to avoid the consequences of HS.

## References

[B1] Liberman M Curr Opin Crit Care 2007 13 691 696 10.1097/MCC.0b013e3282f1e77e 17975392

